# Optimal combination therapy for metastatic hormone-sensitive prostate cancer: new evidence, challenges and unanswered questions

**DOI:** 10.1097/MOU.0000000000001124

**Published:** 2023-09-11

**Authors:** Fabio Zattoni, Pawel Rajwa, Giorgio Gandaglia

**Affiliations:** a Department of Surgery, Oncology, and Gastroenterology, Urology Clinic, University of Padua, Padua, Italy; b Medical University of Silesia, Zabrze, Poland; c Department of Urology, Comprehensive Cancer Center, Medical University of Vienna, Vienna, Austria; d Unit of Urology/Division of Oncology, Urological Research Institute, IRCCS San Raffaele Hospital, Milan, Italy

**Keywords:** metastases-directed therapy, metastatic hormone-sensitive prostate cancer, prostate cancer, treatment intensification, triplet therapy

## Abstract

**Purpose of review:**

To evaluate the evidence supporting treatment intensification in mHSPC, with a focus on possible indications for treatment in each clinical setting.

**Recent findings:**

There is a growing armamentarium of treatment options for patients with metastatic hormone-sensitive prostate cancer (mHSPC). These include combinations of treatments such as androgen deprivation therapy (ADT), docetaxel, and new antiandrogenic therapies. Treatment intensification with chemotherapy or newer hormonal agents may improve patient's oncologic outcomes, but it can also come with additional toxicities and costs. Therefore, we need to take into account individual patient factors and preferences when deciding on the optimal combination therapy. Additionally, ongoing research is needed to identify biomarkers and new image techniques that can predict response to treatment and identify the best candidate for each treatment.

**Summary:**

Challenges and unanswered questions regarding treatment intensification and de-intensification are still present. Further studies are still needed to identify which patients would benefit most from this approach to improve quality of life without compromising overall survival outcomes.

## INTRODUCTION

The standard of care (SOC) for mHSPC has traditionally been androgen deprivation therapy (ADT) alone, but several clinical trials have demonstrated that the combination of chemotherapy and/or novel androgen receptor targeting agents (ARTA) in addition to ADT can improve oncologic outcomes [1].

According to EAU and NCCN guidelines, the SOC for PCa is treatment intensification using doublet or triplet therapy [2]. Despite this recommendation, data from the Veterans Affairs healthcare system, as well as from Medicare claims data suggests that majority of men are still treated with ADT alone for mHSPC [3]. The reasons for this could be a financial burden, nonadherence to guidelines, and old treatment habits. Although there is recent interest in treatment de-intensification for certain patient populations to decrease toxicity and enhance quality of life, the identification of the optimal candidate for the right combination therapy at the right time represents an unmet need in the metastatic PCa setting. This narrative review summarizes the most relevant published data to guide a multidisciplinary team in selecting appropriate candidates for treatment intensification in mHSPC. 

**Box 1:**
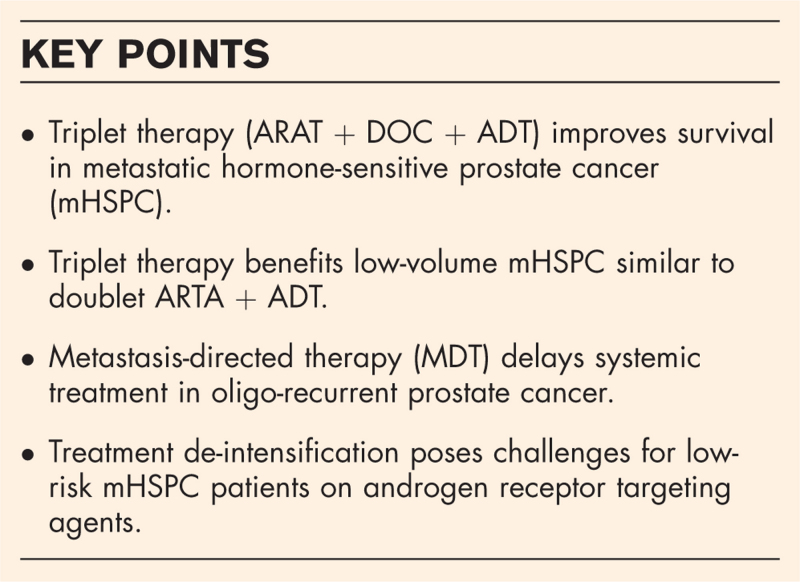
no caption available

## EVIDENCE ACQUISITION

A literature review identified recent studies on treatment intensification and de-intensification for mHSPC. PubMed database was used, and the collected studies formed the basis for a narrative analysis of the literature published in the last 18 months.

## TREATMENT INTENSIFICATION AND TRIPLET THERAPY

Two randomized controlled trials, namely PEACE-1 and ARASENS, showed in 2022 that triplet therapy (ARAT [PEACE-1: abiraterone + DOC + ADT; ARASENS: darolutamide (DARO)] + DOC + ADT) offers an OS advantage compared to doublet therapy (DOC + ADT) in mHSPC setting. This underscores the importance of early addition of ARTA [4^▪▪^,^5^].

In the phase 3 PEACE-1 trial [4^▪▪^,^6^], patients with de novo mHSPC were enrolled using a 2x2 factorial design. Participants had to comply with continuous and on-study ADT, and those receiving ADT for up to 3 months were eligible. The study randomly assigned patients in a 1 : 1:1 : 1 ratio to receive SOC with DOC and ADT, SOC with abiraterone, SOC with radiation, or SOC with both radiation and abiraterone. The study had a good balance of disease and demographic factors. Among the 710 patients who received ADT plus DOC, the median age was 66 and approximately 78% had a Gleason score greater than 8. Most patients (80%) had metastasis only in the bones, whereas 8% had metastasis in the lymph nodes and around 12% had visceral metastasis. Of the patients, 36% had low-volume disease and 64% had high-volume disease per CHAARTED criteria. The primary endpoints were radiographic progression-free survival (rPFS) and OS. The addition of abiraterone to ADT and DOC resulted in a significant improvement in median rPFS compared to SOC alone (HR, 0.50; *P* < 0.01). The SOC plus abiraterone group also experienced improved OS compared to the group without abiraterone, both in the overall population and in the population of patients receiving ADT and DOC [18,19]. The ARASENS trial [5,7], tested the triplet combination of ADT + DOC + DARO versus the DOC doublet in newly diagnosed mHSPC patients with mostly de novo mHSPCA and high-volume disease. DARO demonstrated a significant 32.5% reduction in the risk of death compared to the control regimen. Additionally, DARO showed longer time to castration-resistant disease and prolonged time to pain progression compared to placebo. These improved outcomes were associated with only a modest increase in adverse events. The trial results consistently demonstrate the benefit of adding an AR-targeted agent onto an ADT/ DOC backbone, with a clear and early separation of curves indicating an overall survival benefit in the investigative arm (HR 0.675, 95% CI 0.568–0.801; *P* < 0.0001). Importantly, for both trials, these improved outcomes of triplet therapy intensification were associated with only a modest increase in adverse events. mHSPC can be categorized into two subtypes based on disease spread and risk level. The STOPCAP M1 collaboration conducted a meta-analysis of individual participant data from GETUG-15, CHAARTED and STAMPEDE trials to investigate the effectiveness of adding DOC to ADR in mHSPC [8]. The analysis demonstrated the clear benefits of DOC in terms of OS, PFS, and failure-free survival. The effect of DOC on PFS varied depending on the volume and timing of metastatic disease. Docetaxel improved PFS and OS in most men, except those with low volume, metachronous disease. Patients with high volume synchronous metastatic disease experienced a significant 11% absolute improvement in OS (from 25% to 36%), while those with low volume synchronous metastatic disease had a smaller 6% absolute improvement in OS (from 55% to 61%). These findings suggest that men with low volume, metachronous disease should be managed differently compared to those with other types of metastatic disease.

A recent network Meta-analysis (NMA) [9^▪^] showed that for low-volume mHSPC with ADT as the reference, the benefit of combination therapies, apart from ARAT + ADT, was not significant. When doublet ARAT + ADT therapies were pooled as the NMA reference, as these doublets are the current “standard of care” in many countries, and triplet therapy had to compete with them in low-volume mHSPC, there was no significant difference in OS for DARO + DOC + ADT (hazard ratio [HR] 1.04, 95% confidence interval [CI] 0.58–1.87;) or abiraterone + DOC l + ADT (HR 1.27, 95% CI 0.70–2.28;) compared to the ARAT + ADT. The triple therapy consisting of DARO, DOC, and ADT, as well as the one comprising ABI, DOC, and ADT, were linked to enhanced OS in contrast to doublet therapy. However, they showed no significant improvement compared to the ARTA doublets. Among patients with high-volume disease, the ABI triplet may increase OS compared to DOC doublet, but not when compared to other ADT-based treatments or ABI doublets. In the case of low-volume disease, the ABI triplet may not enhance OS compared to other ADT-based treatments. The report highlights the need to consider disease volume and doublet comparisons when assessing the benefits of triplet therapy. Ultimately, the study concludes that there is no clear preference between triplet regimens and ARTA [10^▪▪^].

Recently, the ARASENS and ENZAMET trial released data regarding the efficacy and safety of the treatment for subgroups based on disease volume and risk. The ARASENS trial showed that patients with high-volume, high-risk, and low-risk disease who received treatment intensification with DARO+ADT+DOC experienced increased overall survival compared to those who received placebo+ADT+DOC. Additionally, the results were suggestive of a survival benefit in the smaller low-volume subgroup. The study also found that there was a benefit in time to castration-resistant prostate cancer and subsequent systemic antineoplastic therapy across all disease volume and risk subgroups. Moreover, the subgroup analysis demonstrated a similar adverse event profile consistent with the overall population [11^▪▪^]. The ENZAMET trial measures the long-term OS using ENZA + ADT, compared to first-generation androgen receptor inhibitor. ENZA+ plus first-generation androgen receptor inhibitor without DOC improved overall survival for patients with synchronous and metachronous mHSPC. Adding ENZA to ADT consistent clinical benefits across most prognostic subgroups and supports previous findings that adding effective ARTA+ ADT+ DOC benefits patients with synchronous mHSPC [12^▪▪^].

## TREATMENT INTENSIFICATION AND TRIPLET THERAPY: IMPLICATIONS FOR CLINICAL PRACTICE

It is important to note that treatment intensification comes with additional toxicities and costs. Careful consideration of individual patient factors, such as age, comorbidities, patient preferences, and counseling on possible side effects, is necessary when deciding on treatment intensification.

Both young and old mHSPC patients benefit from combination systemic therapies, with no significant difference in benefits based on age. However, younger patients seem to benefit more from ARTA plus ADT, while triplet therapy (ADT plus DOC plus ARTA) has the highest probability of overall survival benefit, but the benefit is less pronounced in older patients [13^▪^].

Upfront DOC is a cost-effective option for eligible men with mHSPC per a Japanese study with 340 patients treated with either upfront DOC or upfront ABI. Though there was no significant difference in PFS, the medical costs were lower in the DOC group ($1239) than the ABI group ($3453) [14^▪^].

Regarding HRQoL, treatment with ADT + DOC showed a decrease in general HRQoL and increased fatigue compared to ADT alone and ADT + ABI, while ADT + ABI had a statistically significant, but not clinically significant, improvement in general HRQoL, pain, and fatigue scores compared to ADT alone. No benefit was found for ADT + ENZA, ADT + APA, and ADT + radiotherapy, except for increased fatigue with ADT + enzalutamide compared to ADT alone. Systemic combination treatments showed higher rates of grade 3–5 AEs. ADT + abiraterone, ADT + ENZA, and ADT + APA were likely to provide a net clinical benefit, whereas ADT + DOC and ADT + DOC + DARO were unlikely to be beneficial considering both survival benefits and potential AE [15].

The association between baseline body mass index (BMI) and metformin exposure with quality of life (QOL) and PCa outcomes including survival in patients enrolled in the CHAARTED trial has been evaluated in 788 patients [14^▪^]. Lower BMI was associated with high volume disease and poorer baseline QOL on functional assessment of cancer therapy-prostate. Metformin exposure was not associated with survival in the small exploratory multivariable analysis. Overall, the study did not find a link between baseline BMI and survival, but lower baseline BMI was associated with features of greater cancer burden and poorer QOL [14^▪^].

Therefore, it is essential to correctly inform our patients, particularly if they are considering triplet therapy. Ongoing trials for mHSPC aim to further de-escalate and personalize therapy.

## METASTASES-DIRECTED THERAPY

A metastasis-targeting therapy (MDT) has been proposed with the aim to delay systemic treatment and/or expand treatment landscape of mHSPC [16^▪▪^]. For synchronous metastasis there is no evidence to support MDT. While there are several reasonable hypotheses, only retrospective data are available, and thus there is no high level of evidence. There are two randomized phase II trials conducted for metachronous tumors with oligo-recurrent PCa, namely STOMP [17] and ORIOLE [18], which tested respectively MDT using surgery ± SABR versus surveillance or SABR versus surveillance. By analyzing the results of STOMP and ORIOLE together, the combined results confirmed the significant improvement in PFS in favor of MDT (HR: 0.44, *P* < 0.001) [19].

A recently published EXTEND trial [20^▪▪^] investigated whether the addition of MDT to intermittent hormone therapy improves oncologic outcomes and preserves time with eugonadal testosterone compared to intermittent hormone therapy alone. It included oligo metastatic patients, both synchronous and metachronous. Half of the patients received SBRT and six months of hormone therapy, while the other half received hormone therapy alone. Hormone therapy was then discontinued after six months and resumed upon disease progression. Interestingly, the addition of SBRT to ADT resulted in improved progression-free survival (PFS) compared to hormone therapy alone. One of the predefined key secondary endpoints was eugonadal progression-free survival which was defined as the duration from the time of achieving a eugonadal testosterone level to the point of progression. Additionally, the eugonadal testosterone progression-free survival was noteworthy showing that the addition of temporary ADT to MDT improves eugonadal progression-free survival compared to systemic therapy alone. Patients received six months of ADT, and a significant number of them had testosterone recovery and did not experience any progression. Many patients had complete testosterone recovery and did not progress following SBRT and ADT compared to ADT only.

## ANDROGEN RECEPTOR PATHWAY INHIBITOR IN COMBINATION WITH TREATMENT OF THE PRIMARY TUMOR

In 2018, two trials, HORRAD [21] and STAMPEDE [22], investigated the addition of radiotherapy to ADT in patients with newly diagnosed low volume metastatic disease. HORRAD trial with 432 patients showed no OS benefit with ADT + prostate radiotherapy, while STAMPEDE trial with 2061 patients showed no OS benefit in overall group with ADT + DOC + RT, but RT improved FFS with OS improvement in low metastatic burden group. However, the trial did not include RT + ARTA + RT, and there is no evidence of a difference in time to symptomatic local events or global quality of life [23].

The prospectively planned STOPCAP meta-analysis of these trials [24] and the PEACE-1 trial [25] showed an improvement in biochemical progression and FFS, equivalent to a 10% benefit at 3 years, with a 7% improvement in 3-year survival in men with fewer than 5 bone metastases. However, the lack of a consensus definition for low-volume and oligometastatic disease, the role of more sensitive imaging techniques, and ongoing clinical trials investigating prostate radiotherapy in mHSPC remain issues to be resolved. According to the latest update with a median follow-up of 61 months [23], Radiotherapy to the prostate improves median survival and lowers local progression/side effects with minimal or no side effects. Combining radiotherapy with an ARTA has a biological rationale as RT induces double-strand breaks that upregulate proteins involved in nonhomologous end-joining repair, which is inhibited by ADT. This treatment combination is synergistic and more effective AR inhibition may increase the radiotherapy effect, particularly in patients with bulky primary tumors, improving local control. Limited information exists on Cytoreductive radical prostatectomy (cRP) for low-volume mHSPC. Recent prospective studies suggest cRP is safe and associated with improved oncologic outcomes and local disease control. Acceptable adverse events were reported [26].

No strong evidence supports ARTA+ treatment of the primary + ADT, so clinical decisions must be pragmatic. RT and RP have distinct mechanisms of action, are well tolerated, and may have synergistic effects. Although evidence supporting ADT + RT to the primary and ARTA is low, it is a common practice, according to an APPCC survey [27].

## TREATMENT DE-INTENSIFICATION

A metanalysis by Fallara *et al.*[28] of over 5,000 patients with mHSPC compared the effect of triplet therapy (ADR+ DOC + ABI) to DOC or ABI alone. The analysis showed that triplet therapy was associated with longer OS and PFS compared to DOC alone, but yielded similar OS and PFS compared to ABI alone. Both ABI and triplet therapy were found to be superior to DOC. The PEACE-1 trial [29^▪▪^] investigated the use of abiraterone with ADT in low-risk mHSPC patients and found that abiraterone improved PFS but did not improve OS. Similarly, the ORIOLE trial [19] investigated the use of intermittent ADT in mHSPC patients with low-volume disease and found no difference in quality of life or PFS compared to continuous ADT. Treatment de-intensification in mHSPC may be feasible for patients with low-risk disease or those on AR targeted therapy, but this approach presents potential risks, including disease progression and undertreatment. The PEACE-1 trial showed that abiraterone improved PFS but not OS, indicating that patients may experience a temporary delay in progression. However, reducing chemotherapy use or long-term ADT may reduce toxicities. Metastasis-directed therapy may reset the metastatic disease clock, avoiding the need for intense systemic therapy for some time.

## UNANSWERED QUESTION AND CHALLENGES FOR TREATMENT INTENSIFICATION IN mHSPC

Treatment intensification for mCSPC poses challenges and unanswered questions. The term ‘oligometastatic’ is imprecise and misleading, as patients with low metastatic burden may have unlimited metastases in specific locations. The biology of PCa suggests a continuum, and the optimal treatment strategy is controversial. PSMA PET imaging has higher sensitivity and specificity than conventional imaging, but its role as the primary staging modality is debated among oncological societies. Previous trials relied on conventional imaging, and using PSMA PET scans may have drawbacks, such as: false positives leading to unnecessary invasive procedures, exposing patients to systemic therapy that may not benefit them and depriving patients of local therapy that would benefit them [30]. The lack of biomarkers for decision-making adds to the complexity. The effectiveness of Triplet therapy compared to ADT + ARTA remains unproven, and the role of radiation combined with ARTA in low burden de-novo disease is uncertain. Determining the optimal duration of systemic therapy and managing cardiovascular disease in these patients are ongoing challenges.

Unless new evidence becomes available, Table 1 and Table 2 attempt to synthesize the existing evidence and management options for mHSPC.

**Table 1:** Management of mHSPC: an evolving systematic treatment paradigm

ADT + Docetaxel	ADT ± Docetaxel: GETUG 15, CHAARTED and STAMPEDE ARM C
ADT + Androgen Synthesis inhibitor	ADT ± Abiraterrone + prednisone: LATITUDE and STAMPEDE Arm G
ADT + Potent Androgen Receptor Inhibitor	ADT ± Enzalutamide: ENZAMET and ARCHES ADT ± Apalutamide: TITAN
ADT +Radiation to the primary	ADT ± External beam radiation therapy: HORRAD ADT ± External beam radiation therapy: STAMPEDE
ADT +Docetaxel + ARTA = triplet therapy	ADT + docetaxel ± Abirateron + Docetaxel ± RT: PEACE 1 ADT + docetaxel ± Darolutamide + Docetaxel: ARASENS

**Table 2:** Treatments options according to Metastatic Hormone-Sensitive Prostate Cancer aggressiveness

	Fit^a^ Synchronous M1 ’High-volume and/or “high risk’	Fit^a^ Synchronous M1 ’Low-volume “ and/or low risk’	Fit^a^ Metachronous M1 ’High-volume and/or “high risk’	Fit^a^ Metachronous M1 ’Low-volume and/or“low risk’
ADT alone	No	In minority of selected patients/avoid	No	In minority of selected patients/avoid
ADT+Docetaxel	Only selected patients	No	No	No
ADT+Abirateone/P	YES For fit patients, consider triplet therapy	Yes	Yes	Yes
ADT+Enzalutamide				
ADT+Apalutamide				
ADT+Docetazel + Abirateron/P or Darolutamide		Triplet: selected patients	Triplet: selected patients	No
Treatment of the primary tumor	Only in case of local complications	Yes (RT)	Local salvage therapy in selected cases	
Metastases-directed therapy (MDT)	Only in case of local complications	Ideally on clinical trial. Aim: to stop systemic therapy after a fixed duration in case of good response	Only in case of local complications	Ideally on clinical trial. Aim: to avoid systemic therapy or to stop systemic therapy after a fixed duration in case of good response?

a Frail/vulnerable patients: individualized treatments recommendation. M1 assessment: On bone scintigraphy and computed tomography.

## CONCLUSION

Adding chemotherapy or newer hormonal agents has improved outcomes in mHSPC. Treatment decisions should be personalized, considering individual patient factors and preferences. Treatment de-intensification may be viable for some patients, but further research is needed to identify the appropriate population. Clinicians should weigh the potential benefits and risks of treatment combinations carefully. The ultimate goal is to maintain quality of life and provide maximal overall survival. More research is needed to identify biomarkers for treatment selection.

## Acknowledgements

*None.*

### Financial support and sponsorship

*None.*

### Conflicts of interest

*No conflicts to declare.*

## REFERENCES AND RECOMMENDED READING

Papers of particular interest, published within the annual period of review, have been highlighted as:

▪ of special interest
▪▪ of outstanding interest
